# Using Social Media to Generate and Collect Primary Data: The #ShowsWorkplaceCompassion Twitter Research Campaign

**DOI:** 10.2196/publichealth.7686

**Published:** 2018-04-23

**Authors:** Wendy Clyne, Sally Pezaro, Karen Deeny, Rosie Kneafsey

**Affiliations:** ^1^ Hope for the Community, Community Interest Company Coventry United Kingdom; ^2^ School of Nursing, Midwifery and Health Coventry University Coventry United Kingdom

**Keywords:** work engagement, health personnel, empathy, attitude of health personnel

## Abstract

**Background:**

Compassion is a core value embedded in the concept of quality in healthcare. The need for compassion toward healthcare staff in the workplace, for their own health and well-being and also to enable staff to deliver compassionate care for patients, is increasingly understood. However, we do not currently know how healthcare staff understand and characterize compassion toward themselves as opposed to patients.

**Objective:**

The aim of this study was to use social media for the generation and collection of primary data to gain understanding of the concept of workplace compassion.

**Methods:**

Tweets that contained the hashtag *#ShowsWorkplaceCompassion* were collected from Twitter and analyzed. The study took place between April 21 and May 21, 2016. Participants were self-selecting users of the social media service Twitter. The study was promoted by a number of routes: the National Health Service (NHS) England website, the personal Twitter accounts of the research team, internal NHS England communications, and via social media sharing. Participants were asked to contribute their views about what activities, actions, policies, philosophies or approaches demonstrate workplace compassion in healthcare using the hashtag #ShowsWorkplaceCompassion. All tweets including the research hashtag #ShowsWorkplaceCompassion were extracted from Twitter and studied using content analysis. Data concerning the frequency, nature, origin, and location of Web-based engagement with the research campaign were collected using Bitly (Bitly, Inc, USA) and Symplur (Symplur LLC, USA) software.

**Results:**

A total of 260 tweets were analyzed. Of the 251 statements within the tweets that were coded, 37.8% (95/251) of the statements concerned Leadership and Management aspects of workplace compassion, 29.5% (74/251) were grouped under the theme related to Values and Culture, 17.5% (44/251) of the statements related to Personalized Policies and Procedures that support workplace compassion, and 15.2% (38/251) of the statements concerned Activities and Actions that show workplace compassion. Content analysis showed that small acts of kindness, an embedded organizational culture of caring for one another, and recognition of the emotional and physical impact of healthcare work were the most frequently mentioned characteristics of workplace compassion in healthcare.

**Conclusions:**

This study presents a new and innovative research approach using Twitter. Although previous research has analyzed the nature and pattern of tweets retrospectively, this study used Twitter to both recruit participants and collect primary data.

## Introduction

### Using Twitter for Health-Related Research

An increasing number of studies have used Twitter for health-related research. Twitter has been used by researchers not just as a mechanism for dissemination of research findings [1,2] but also as a source of data that is available in the public domain [3]. Research has been particularly fruitful in the areas of public health and infectious diseases where Twitter has been used, for example, to track geographically reports of illness related to consumption of restaurant foods [4] and to explore whether Twitter can be an effective tool for the surveillance of communicable diseases spread at large festival and public gatherings [5]. Both the pattern and the content of tweets about influenza during the H1N1 pandemic were examined to explore how Twitter is used to share health information [6]. The authors highlight the potential for Twitter-based research to aid our understanding of the predictors of uptake of health interventions, such as vaccination, following public health campaigns. The content of tweets was also examined in a study concerned with tweet content in the day-to-day Twitter communications of health professionals [7].

Researchers have also used online social networking sites as a mechanism for recruiting participants to traditional research studies. Online recruitment has been used for recruiting underserved and hard-to-reach groups that may be difficult to engage by more traditional means [8]. Research studies using this approach have conducted online research recruitment campaigns across a number of online platforms simultaneously (Facebook, Twitter, LinkedIn) [8] or across a single platform (Twitter) [9,10] to recruit participants to take part in research using traditional data collection methods. For the Twitter component of their multiplatform recruitment strategy, Yuan et al [8] created a research account and associated hashtags to recruit individuals to an online survey about HIV clinical outcomes. They found social media to be indispensable for recruitment and were particularly taken with the efficiency and cost-effectiveness of their approach.

### Crowdsourcing Primary Data

In our study, we combined the benefits of Twitter as a participant recruitment method and also used Twitter as a means for generating primary data. To the best of our knowledge, this is the first study to both recruit participants and collect primary data using Twitter.

Our study concerned compassion in healthcare. Compassion for patients is a central tenet of the delivery of care and treatment to patients to prevent and treat ill-health. Compassion has been defined as “the combination of underpinning emotions (such as sympathy and empathy), with altruistic values, (particularly a desire to help others), which together motivate an individual to take action, which would ultimately be experienced as ‘care’ by the recipient” [11]. Health staff who show compassion toward patients tend to be more effective at delivering care [12]. Latterly, the concept of compassion has been applied to the way in which healthcare is designed and delivered at the organizational level [13]. Thus, the recipient of compassion in healthcare may be staff, not just patients. Given that healthcare employers have a responsibility to ensure the health and safety of their staff, and the evidence that positive staff experience is associated with positive patient experience [14], compassion toward staff potentially benefits both staff (directly) and patients (indirectly). Although the concept of compassion toward patients has been explored and defined, we do not, as yet, understand the nature and characteristics of compassion as it applies to healthcare staff. In our study, which was conducted using Twitter, we invited healthcare staff to tell us what #ShowsWorkplaceCompassion. Twitter users working in any role, setting, or organization in healthcare across England were invited to participate in a research campaign to explore the characteristics of workplace compassion as they perceived it.

## Methods

### Design

This qualitative study reports the analysis of tweets posted by users of the online social media platform Twitter and uses template analysis to explore what Twitter users perceive shows workplace compassion in a healthcare setting. Statistical data were also captured about the frequency, nature, and origin of online engagements with the study.

### Participant Recruitment

A study webpage was uploaded to the National Health Service (NHS) England website. This invited people to participate in the research study as follows: “We want to know what you think a ‘compassionate healthcare organisation’ looks like. It’s easy to take part. Just tweet your views about what activities, actions, policies, philosophies, approaches, that demonstrate that a healthcare organisation is compassionate using the hashtag #ShowsWorkplaceCompassion. Your contributions—your tweets—might be based on your own experiences of working in healthcare, your experience of providing or commissioning healthcare or based on your general views about what healthcare organisations are about. There are no right or wrong ideas or perspectives, it is your ‘vision’ we are interested in.” Potential participants were advised that they could tweet as many times as they chose and were given a reminder to always use the hashtag #ShowsWorkplaceCompassion. A link was provided to a participant information leaflet. This was shared throughout the data collection period by the research account (@NHSStaffExp) and the research team. The research study was promoted on social media from the individual Twitter accounts of the research team and the NHS England Twitter account. Key influencers on Twitter were contacted ahead of study launch and invited to promote the study. Key influencers included prominent online health and social care individuals and organizations such as the “WeCommunities,” patient groups, research centers, NHS organizations, healthcare commentators, commissioners, and healthcare leaders. Key influencers were told about the purpose of the study and how we envisaged the hashtag would be used by participants:

In a few weeks time, we will be launching a twitter account and the hashtag #ShowsWorkplaceCompassion. We will be inviting people to tweet what they think shows workplace compassion for staff by tweeting sentences that end with #ShowsWorkplaceCompassion (e.g., ‘letting me leave early when my son was ill #ShowsWorkplaceCompassion’; ‘senior management talking to staff #ShowsWorkplaceCompassion’, or ‘I know my manager cares about me and that #ShowsWorkplaceCompassion’. We do hope you will join us in finding out and tweeting about what #ShowsWorkplaceCompassion.

Throughout the data collection period, the research account tweeted daily to encourage Twitter users to participate in the study. These tweets targeted Twitter users working in healthcare. Promotional tweets were also used to recruit participants. Each promotional tweet was seen by between approximately 1100 and 4300 Twitter users. Links to the study webpage were also shared via other social media platforms.

### Procedure

Before data collection, the research hashtag “#ShowsWorkplaceCompassion” was registered with Symplur (Symplur LLC, USA) as a healthcare hashtag to monitor the frequency and number of tweets shared and seen by Twitter users. The URL links to both the study information webpage and the participant information webpage were also logged via Bitly (Bitly, Inc, USA) software to record the geographical location and number of online engagements with both webpages.

The Twitter research account was monitored between 8 am and 11 pm (GMT) for 7 days a week over the 4-week study period in Spring 2016. The biographical information for this research account was listed as “Research account: (@CovUni_CTEHR & @NHSEngland). Let us know what #ShowsWorkplaceCompassion. tweets used for research. See full details→URL Link.” URL links to both the study information webpage and the participant information leaflet webpage were visible at all times on the research account’s profile page.

Hootsuite software was used to schedule and send 9 tweets from the research account between 6 am and 10 pm each day over the period of data collection. Tweets sent by the research account such as “RU a healthcare worker? Have you just finished a nightshift? Did you experience anything that #ShowsWorkplaceCompassion? Let us know! Pls RT” were designed to be promotional and engaging in nature. Links to both the study information webpage and the participant information webpage were also shared.

The permanent “pinned” tweet that remained pinned to the top of the research account’s Twitter page throughout the data collection period was “Our #ShowsWorkplaceCompassion research explores staff ‘visions’ of compassionate healthcare workplaces. See here → URL Link.” The research account also published other tweets on an impromptu basis to answer specific questions and to offer guidance as necessary in response to questions.

Twitter accounts related to healthcare were “followed” by the research account. This account also “liked” and “retweeted” (shared a copy of) every tweet that contained the hashtag “#ShowsWorkplaceCompassion.” At the end of the data collection period, the pinned tweet on the research account was changed to “Data collection 4 #ShowsWorkplaceCompassion project has finished→ URL Link Thx 2 all participants. Results 2B published here.”

### Data Collection

Qualitative data were collected via tweets posted on Twitter. Statistical data about the nature, frequency, number, and geographical location of research related to online engagements were downloaded using Symplur and Bitly software. All tweets posted during the 4-week data collection period containing the hashtag #ShowsWorkplaceCompassion were downloaded directly from the Twitter platform into Microsoft Excel.

### Data Analysis

Symplur software was used to identify the frequency and number of tweets seen and shared by Twitter users. The geographical location and number of known online engagements with both the study information webpage and the participant information webpage were identified using Bitly software.

The downloaded tweets were initially sorted into chronological order to allow the identification and removal of any duplicated material. Any tweets originating from either the research team or the research account were also excluded from the dataset. Additionally, tweets that simply promoted this research or referred the research team to secondary data sources were also removed at this point.

A total of 110 tweets (12.9%) that included the research hashtag expressed support for the research project and encouraged other followers to share their ideas. Although these were considered to contribute to the success of this research, they were not suitable for analysis and were therefore excluded from the analysis. Of the remaining tweets, 8.8% (n=22) either partially or completely referred to patients as the target of compassion rather than staff. These references were therefore excluded from the analysis. While 1.8% (n=16) of tweets containing the research hashtag directed the research team toward secondary sources, 1.4% (n=12) of tweets were retweets (shared copies) of other tweets. These tweets were also excluded from the analysis.

Subsequently, content analysis was conducted for each tweet. An adapted template analysis approach was used [15]. The coding scheme from a focus group about workplace compassion with healthcare commissioners (reported elsewhere) was used as the basis for analysis of data in this study. Four themes—(1) feelings, (2) actions, (3) circumstances, and (4) organizational factors—served as the template for analysis. As the analysis progressed, the template was adapted to the emerging data with theme names changed to “Values and Culture,” “Activities and Actions,” “Personalized Policies and Procedures,” and “Leadership and Management.” All content analysis was conducted by 1 coauthor (SP) and then checked and confirmed by a second coauthor (WC). Areas of disagreement between reviewers were resolved by consensus on a case-by-case basis. Reviewers were blind to identifying information about participants during content analysis.

Statements that described feelings such as having a common purpose or feeling valued as incidences of workplace compassion were coded under the appropriate subtheme within the first theme (Values and Culture). Any actions described as incidences of workplace compassion within statements were coded under the appropriate subtheme within the second theme (Activities and Actions). Individual circumstances covered by policies and procedures that were perceived to show workplace compassion were coded under the appropriate subtheme within the third theme (Personalized Policies and Procedures). Statements that described the wider organizational, practical, and/or leadership principles as instances of workplace compassion were coded under the appropriate subtheme within the final theme (Leadership and Management).

## Results

### Recruitment and Social Media Activity

Following a 4-week data collection period, 850 tweets that included the research hashtag were collected from Twitter. The study webpage was accessed 177 times, and the participant information webpage was accessed 406 times. The participant information leaflet was accessed more frequently than the general study information webpage. Both were accessed more frequently in the first half of the research campaign.

**Figure 1:**
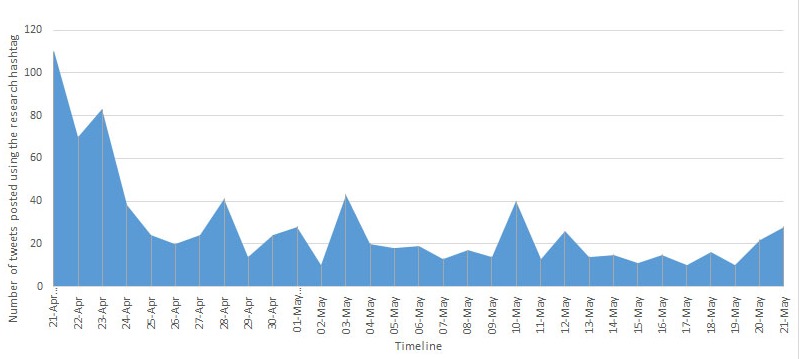
Tweet activity for the #ShowsWorkplaceCompassion hashtag.

The number of tweets posted using the research hashtag over the 4-week data collection period were plotted from week 1 to week 4 (Figure 1). Tweet activity was the highest during the first week of the research campaign and then dropped during the remaining 3 weeks.

Table 1 summarizes the number of tweets that were included in the content analysis and reasons for exclusion, as well as information about recruitment and social media activity data. The research team noted that there were 2 newly created Twitter accounts that had not tweeted before tweeting with the study hashtag. However, the majority of tweets were posted by established Twitter users. The research hashtag made 6,037,026 online impressions, that is, the research hashtag was delivered to Twitter users’ Twitter streams 6,037,026 times. A total of 452 tweets were generated by the research team or the research account to promote the study, resulting in generation of 260 tweets that were appropriate for content analysis.

### Content Analysis

Content analysis was used to analyze the 260 tweets concerning what shows workplace compassion in healthcare. This process resulted in the development of 4 themes and 19 subthemes (Table 2).

Of the 251 statements within the tweets that were coded, 37.8% (n=95) of statements concerned Leadership and Management aspects of workplace compassion, 29.5% (n=74) were grouped under the theme related to Values and Culture, 17.5% (n=44) of statements related to Personalized Policies and Procedures that support workplace compassion, and 15.2% (n=38) of statements concerned Activities and Actions that show workplace compassion.

### Leadership and Management

Approximately one quarter of statements (n=26) within this theme referred to an embedded organizational culture of caring for one another as a significant feature of workplace compassion. One tweet reflects this description of workplace compassion as “Taking time to understand everyone’s issues and then pulling together.”

An organizational culture of speaking openly to learn from mistakes comprised a subtheme within the Leadership and Management theme. This openness was referred to from both an organizational and individual perspective. On an individual basis, supporting staff to “speak out safely” was tweeted as a demonstration of workplace compassion. In relation to organizational responses to adverse events, organizational learning and openness were considered to show workplace compassion. One tweet captured this view as follows: “Learning from mistakes instead of punishing #ShowsWorkplaceCompassion.” Another tweet captured how “Regulation that engages with the reality not the fantasy #ShowsWorkplaceCompassion.”

**Table 1:** Summary of included and excluded tweets, recruitment, and Twitter activity.

Included and excluded tweets			Number
Total tweets extracted			850
Tweets originating from the research account removed			295
Tweets originating from the research team removed			157
Tweets promoting the research removed			110
Tweets leading toward secondary sources removed			16
Retweets removed			12
Total tweets for analysis			260
**Research study information webpage visits**			177
	**Research study information webpage visitor locations**		
		United Kingdom	106
		Germany	43
		United States	20
		Unknown	8
	**Referring channels to research study information webpage**		
		Unidentifiable	79
		Twitter	66
		Links	25
		Unknown	7
**Participant information webpage visits**			406
	**Participant information webpage visitor locations**		
		United Kingdom	247
		Germany	116
		United States	14
		Unknown	29
	**Referring channels to participant information webpage**		
		Unidentifiable	149
		Facebook	109
		Links	91
		Unknown	57
**Tweet activity for the #ShowsWorkplaceCompassion hashtag**			
	Number of hashtag impressions		6,037,026
	Number of Twitter accounts using the hashtag		645

No blame and no bullying leadership and management were also referred to as characteristics of workplace compassion. Action taken against bullying was seen as characteristic of workplace compassion, as tweets stated how “meaningful action & education to identify & act on bullying #ShowsWorkplaceCompassion” and also the “elimination of bullying and bullies…” The identification of “No belittling of staff” and “No gossiping and no bullying” were also put forward as evidence of workplace compassion. Instead, there was a call to “treat others as we would wish to be treated.”

One statement stating that “leaders must role model positive behaviours and values” is representative of many other statements within this theme, which describes how “Compassion is a key component of leadership” and that “Leadership and the right culture is essential” in the demonstration of workplace compassion. Comparable tweets described workplace compassion as “Starting from the top.” Here, compassionate leadership was seen as “fundamental,” yet it was also recognized that organizationally, all staff “living the values of compassion” showed workplace compassion.

**Table 2:** Themes and subthemes for what #ShowsWorkplaceCompassion.

Theme and subtheme, n (%)		Count
**Leadership and Management, 95 (37.8)**		
	Embedded organizational culture of caring for one another	26
	Speaking openly to learn from mistakes	19
	No blame/no bullying management	18
	Inspiring leaders and collective leadership	14
	Financial investment in staff	10
	Recognize humanity and diversity	8
**Values and Culture, 74 (29.5)**		
	Common purpose in a team	19
	Feeling valued	18
	Being heard	16
	Enjoying work	9
	Engaged	8
	Use of caring language	4
**Personalized Policies and Procedures, 44 (17.5)**		
	Recognition of the emotional and physical impact of healthcare work	22
	Recognition of nonwork personal context	10
	Work/life balance is respected	6
	Respecting the right to breaks	4
	Being treated well when unwell	2
**Activities and Actions, 38 (15.2)**		
	Small gestures of kindness	30
	Provision of emotional support	8

### Values and Culture

The first subtheme comprising the highest number of statements about Values and Culture concerned sharing a sense of common purpose within a team. “Compassionate teams are key. Do we care about, value and respect everyone in the team?” is typical of tweets within this theme, along with “clear values shared by all” and “proud to be part of a team.”

This subtheme was closely followed in frequency by one in which workplace compassion is demonstrated by feeling trusted, valued, appreciated, and respected. Tweets such as “Trusting me to do the right thing #ShowsWorkplaceCompassion” and “Nurturing good people by valuing, respecting, rewarding them #ShowsWorkplaceCompassion.” One tweet also described feeling heard as well as trusted, as “Being trusted and being listened to is important #ShowsWorkplaceCompassion.” Being listened to was reported to show workplace compassion and was expressed in other tweets such as “If [staff] feel heard & empowered they’ll treat others [the] same.”

Following on from this, feeling engaged and able to have influence in the workplace were described as demonstrating workplace compassion. This is illustrated in the following tweet: “staff can have a say in strategy and what staff say can actually change the way the NHS is managed.” Enjoying the workplace was a characteristic of workplace compassion for some participants. Examples of this include “Having fun at work important—#ShowsWorkplaceCompassion” and “laughter... #ShowsWorkplaceCompassion.”

Use of sensitive and caring language was referred to as demonstrating workplace compassion in tweets such as “Remember words can be weapons. A team that avoids belittling language #ShowsWorkplaceCompassion” and “Recognising the importance of language #ShowsWorkplaceCompassion…‘we’ ‘us’ ‘colleagues’ ‘people’ not ‘you’ ‘they’ ‘staff’ ‘human resources’.”

### Personalized Policies and Procedures

This theme concerned the compassionate application of human resources policies and procedures on an individual basis and when appropriate. When circumstances are challenging for staff, the compassionate operation of policies and procedures to adapt to this were given as examples of workplace compassion. Flexible working patterns that support work-life balance, caring for other relatives, family life, and responses to family crises were also cited as a demonstration of workplace compassion. In this context, tweets described how showing an interest in the home lives and circumstances of staff showed workplace compassion: “my team were fab after the death of my husband” and “on the loss of his mum colleagues bought my husband a tree to remember her.”

The recognition of the emotional and physical impact of caring within an organization was also firmly established as characteristic of workplace compassion, with 1 tweet stating that “We need to talk about compassion fatigue and burnout.” Moreover, this recognition was often described as providing time and space to talk and listen. One statement reflects upon this as “space and time for staff to listen and talk about difficult emotions.”

Some tweets described practical ways in which emotional needs may be met. These referred to mindfulness training, chaplaincy, restorative resilience, and the provision of practitioner health programs and Schwartz rounds.

In reference to staff experiencing episodes of ill health, 1 tweet stated that “Supporting colleagues through mental health crisis and recognising that adverse behaviour is not misconduct” showed workplace compassion. Supporting colleagues through a phased return after sickness absence was proposed in another tweet as showing workplace compassion in challenging circumstances. Overall, workplace compassion is described in this subtheme of tweets as generally “recognising what matters to one another,” with a majority of tweets referring to an interpersonal recognition of each other’s personal circumstances.

### Activities and Actions

A total of 30 statements in one subtheme concerned general pleasantries or small acts of kindness. Examples of these were given in tweets such as “asking how are you today especially to junior staff and a smile goes a long way (#ShowsWorkplaceCompassion)” and “A moment to make a cup of tea, to share a difficult time #ShowsWorkplaceCompassion.” Other tweets referred to other small acts of kindness, such as “a note telling me to stay strong and some flowers” and described acts such as this as “making a big difference.”

## Discussion

### Principal Findings

The 260 tweets analyzed in this study describe the characteristics of workplace compassion for healthcare staff, according to Twitter users participating in this study. Workplace compassion can be seen in Values and Culture, Activities and Actions, Personalized Policies and Procedures, and Leadership and Management. Content analysis showed that small acts of kindness, an embedded organizational culture of caring for one another, and recognition of the emotional and physical impact of healthcare work were the most frequently mentioned characteristics of workplace compassion in healthcare.

### Implications for Healthcare Staff and Organizations

Twitter users were able to describe and convey the characteristics of workplace compassion for healthcare staff within the confines of the 140-character limit of Twitter. This study provides evidence about the characteristics of compassion as it applies to healthcare staff as recipients of compassionate behavior. Future research could usefully explore the extent to which healthcare staff perceive themselves to experience workplace compassion, as characterized here, and whether this varies across types of organizations and staff roles. Given the interplay between staff and patient experience, the experience of workplace compassion may be particularly key for frontline staff [16,17].

The Values and Culture that reflect workplace compassion were clearly articulated by participants in this study: a sense of common purpose with others, having a voice and being heard, and feeling valued in the workplace.

The research presented here potentially informs actions by healthcare organizations to improve the experience of staff and the ways in which healthcare organizations measure staff experience. Compassionate leadership, defined as leadership that is adaptive, shared, and distributed [13], has been the focus of research concerned with healthcare staff as recipients of compassion. Participants in this study confirm the importance of leadership and management in achieving compassion in the workplace, with leadership and management comprising the largest category of tweets. As a consequence of this study, healthcare leaders and managers know that compassionate leadership can be achieved by supporting staff to care for one another, taking action against bullying, creating a learning culture, and inspiring others as a role model of compassion in the workplace.

For many participants in this study, workplace compassion is embodied in the interpersonal activities and actions of colleagues, from taking the time to make a drink for a colleague to appreciation of the wider context of a staff member’s personal circumstances. In this regard, compassion toward staff, similar to compassion toward patients, has a significant interpersonal behavior component [18]. When staff surveys (such as the NHS staff survey) do cover interpersonal actions and behaviors, these tend to focus on the actions of immediate managers and senior managers rather than peers and colleagues in the workplace. In so doing, such surveys fail to capture a significant interpersonal element of staff experience. Although leadership is clearly an important element of workplace compassion, this study indicates that staff surveys should be adapted to capture the activities and actions that take place between colleagues to fully capture the range of actors involved in achieving workplace compassion and positive staff experience.

Although many health and care staff gain satisfaction from the provision of compassionate care, it can also be emotionally, physically, and mentally demanding for staff. Errors in health and care also take a toll on health and care staff in addition to the impact that errors have on patients and their families. The term “second victim” is sometimes used to describe the impact on the workforce when harm occurs to patients in the receipt of healthcare services. A characteristic of workplace compassion identified in this study is the application of policies and procedures to individuals in ways that acknowledge this reality for staff.

For participants in our study, workplace compassion exists in the details of policies and procedures but is enacted interpersonally between staff, and not solely in interactions with staff in hierarchical positions of authority and power. Initiatives that support healthcare staff to be compassionate toward and between their colleagues in the ways described by participants in this study, including at times of work pressure and when staff have experienced an emotional and physical impact of their work, could be a more effective way to increase the experience of workplace compassion than the development of workplace policies and statements extolling the virtues of compassion in the workplace. As we know that organizations that prioritize staff health and well-being perform better, with improved patient satisfaction, stronger quality scores, better outcomes, higher levels of staff retention, and lower rates of sickness absence [19], and that healthcare organizations can take action that improves support for staff and staff well-being [20], an understanding of the characteristics of workplace compassion is key to making the organizational and cultural change necessary.

### Methodological Issues

Our study, which took place over a 1-month period, saw 645 Twitter accounts participate in the #ShowsWorkplaceCompassion research hashtag, generating 260 tweets that could be analyzed to answer our research question. Previous research has compared the efficiency of Web-based recruitment and data collection with paper-based and postal methods. Ebert et al [21] found that Web-based recruitment and data collection generated more complete data and was more cost-effective than postal recruitment and paper based data collection by a factor of 10. Processing digital questionnaires was half the cost of handling postal questionnaires. A randomized controlled trial of recruitment and data collection methods for a survey of doctors found that Web-based methods were cheaper than combined Web-based and postal methods, with no evidence of increased response bias but lower response rates [22]. This study offers similar cost efficiencies through Web-based data collection. Tweets from the research account were automated and scheduled, and daily monitoring of hashtag use and retweeting of contributions to the study could easily be fitted around other research activities.

Previous studies with Web-based data collection recruited potential participants digitally using existing email distribution lists. In contrast to previous studies, this study enabled open recruitment of participants for whom no contact details were available with the research team. Twitter provides the ability to crowdsource primary data. However, the ease and openness of this method of data collection is countered by a challenge to the generalizability of our findings, given that participants were existing Twitter users willing to respond to the research campaign and thus highly self-selecting. Researchers considering using this method need to consider the trade-off between these factors when selecting their methodological approach.

Twitter offers the additional benefit to participant recruitment of easy reach across a wide geographical area. The study webpage was visited by a sizable minority of international participants, despite active promotion only within England. Future research studies seeking an international dataset may find that using Twitter as a recruitment and data collection medium in itself promulgates the research study. Twitter offers a particularly viable route to participant recruitment and primary data collection for research studies with an international focus.

Participation in the study decreased rapidly after the first week of the data collection period. Promotional activity at the start of the study was successful in driving visits to the research account and generating data. Additional promotional activity in later weeks may have been beneficial for maintaining recruitment and momentum. Scheduled tweets from the research account alone were insufficient to maintain the initial level of data collection.

An alternative option for data collection would have been to set up a Twitter chat for data collection. Akin to a focus group, a Twitter chat is a focused discussion of a particular pre-arranged topic or topics to which Twitter users are invited to contribute. Tweet chats often last about an hour. Chai et al [23] tested this approach for primary data collection and found the use of an existing Twitter chat forum essential for securing participants. However, in our study, we were not interested specifically in interaction between Twitter users. Indeed, we conducted a traditional focus group before this study to assist us to formulate this research study, and thus, a Twitter focus group would have duplicated this effort rather than extend our understanding of the topic. Our approach did not rely upon an existing forum of participants active in an existing Twitter chat, but instead sought participants from a wider, open base across Twitter.

The application of formal ethical approval processes and permissions in research using Twitter is variable. In their systematic review, Sinnenberg et al [3] report that about a third of published research studies refer to ethics board approval. As much as Twitter research uses retrospective analysis of existing information already in the public domain, researchers have not necessarily needed to seek formal ethical approval. Although there may be some ambiguity about the extent to which tweets and other social media communications exists in the public versus private domain, and thus the ethical issues that pertain to their use in research [24], there was no such ambiguity in our study. Twitter users were explicitly invited to tweet to generate primary research data in our study. We therefore did seek and secure institutional ethical approval for our approach to participant recruitment. The nature of Twitter does impose several limitations on researchers that necessitates a creative response to ensure consent. As communication on Twitter was limited to 140 characters per tweet, and so participant information details cannot easily be tweeted, we opened a specific account on Twitter for research purposes, and for this study, to be able to share links to the study participant information sheet, hosted elsewhere, in the Twitter account biography and header. The research account was also able to tweet the link to the study participant information routinely at intervals during the data collection period. As the account was new and not a pre-existing Twitter account repurposed for this study, we can be reasonably sure that the research purpose of the account, the hashtag, and the tweets using the hashtag were clear to potential and actual participants. The frequency with which the participant information webpages were accessed is reassuring that many potential study participants did indeed access this information. However, we cannot determine whether all study participants accessed this information. The creation of a specific Twitter account has the additional benefit that we can easily use the Twitter account to share the results of the study with participants and other people that followed the account. The account currently has just under a thousand followers, and we can tweet the details of this research paper directly to those interested in the research.

### Conclusions

Twitter can be an effective means for the recruitment of research participants and the collection of primary data about health-related matters. Our study recruited healthcare staff who use Twitter to describe the characteristics of workplace compassion. We found that small acts of kindness, an embedded organizational culture of caring for one another, recognition of the emotional and physical impact of healthcare work, feeling valued, a sense of common purpose within a team, and being able to speak openly to learn from mistakes characterized compassion toward healthcare staff in the workplace. The need to capture effectively the interpersonal elements of workplace compassion in the measurement of staff experience is discussed, for organizations to seek to achieve positive staff experience in healthcare workplaces.

## Abbreviations

NHS: National Health Service
